# From collective restriction to critical action: the indirect effects of critical motivation and radical hope

**DOI:** 10.3389/fpsyt.2026.1748499

**Published:** 2026-04-27

**Authors:** Radia DeLuna, Helen A. Neville, Tuyet-Mai Ha Hoang

**Affiliations:** 1 Educational Psychology, University of Illinois at Urbana-Champaign, Champaign, IL, United States; 2 School of Social Work, University of Illinois at Urbana-Champaign, Champaign, IL, United States

**Keywords:** collective autonomy restriction, critical consciousness, mental health, radical hope, Women of Color

## Abstract

**Introduction:**

Historically, Women of Color (WOC) in the United States have experienced systemic restrictions to their freedom and autonomy, which can have a lasting impact on their mental health and wellbeing. Conceptually, this type of collective autonomy restriction (CAR) experience may be associated with increased critical consciousness (CC), reflected in greater awareness of social and systemic oppression, commitment to and belief in one’s capacity to address social issues, and engagement in action; however, there is a dearth of research examining this association. Building on critical consciousness and hope literatures, we hypothesized that the association between CAR and critical action would be explained through serial pathways of increased critical motivation and greater radical or collective hope.

**Materials and Methods:**

A sample of 408 WOC completed an online survey administered through Prolific and hosted on Qualtrics. The survey included indicators of CAR, critical consciousness (critical motivation and critical actions), psychological hope, and radical hope.

**Results:**

We conducted structural equation modeling to test a serial mediation model exploring the associations among CAR, critical motivation, hope, and critical action. Findings indicated the association between CAR and critical action was fully mediated by the proposed serial mediation pathways (CAR → Critical Motivation → Radical Hope → Critical Action). The pathway through radical hope was stronger than through psychological hope. The direct effect of CAR on critical action was non-significant, indicating full mediation.

**Discussion:**

These results highlight the role of radical hope as a potential pathway connecting critical awareness of collective autonomy restriction and critical motivation to engage in critical action aimed at social change. We extend the existing literature by demonstrating that awareness of oppression and motivation alone may be insufficient to explain the link between the first two dimensions of critical consciousness (critical reflection and critical motivation) and critical action. Limitations and implications for research and practice are discussed.

## Introduction

The denial of the right to move and engage freely with one’s own cultural group has been used as a tool to limit access and resources for historically excluded communities for centuries. In the colonial United States (U.S.), enslaved African and Indigenous communities were murdered en masse, the living were denied the right to engage in their cultural practices, denied access to their land, forced into labor, and more (1, 2). The atrocities faced by Women of Color (WOC) are uniquely cruel. From the brutalization, rape, and sterilization of the Indigenous and enslaved African women, to the recent criminalization of abortion, and the skyrocketing maternal mortality rates for Black and Native American birthing people (3). Across history, WOC have resisted these forms of violence and demanded autonomy over their bodies and futures. An understanding of the role of oppression in one’s life has been found to serve as a buffer for Black and Latinx communities (4, 5).

Recently, psychologists have begun to explore contemporary forms of gendered harms against women, the ways in which women resist these harms, and how these harms may be associated with awareness of systemic oppressions. One form of harm is restrictions to collective or group autonomy (e.g., restrictions to safe abortion and gender affirming care). Importantly, there is little empirical research exploring the impact of collective autonomy restriction on WOC and their resistance to such injustices (6). Emerging literature has highlighted the role of hope as a catalyst for resistance in the face of oppression; even when circumstances appear bleak, hope fosters a belief in the possibility of a better future, motivating individuals to engage in transformative efforts (7, 8). Research has also sought to identify how experiencing systemic harms (such as racism) may relate to one’s ability to identify systemic power imbalances and understand their role in society (e.g., critical consciousness raising). In this study we built on the extant research by exploring the link between collective autonomy restrictions (CAR) and critical consciousness among WOC; our goal was to advance the work by exploring pathways that may help illuminate the association between CAR and critical consciousness, and whether collective hope can serve as an intervention factor. Specifically, we focused on hope as this explanatory variable given its evident role as protective factor against harm and as a potential catalyst for change (9, 10). We were interested in two dimensions of hope: the traditional psychological notion that captures individual hope and collective hope that focuses on a better future for one’s group. CAR is more closely aligned with collective hope, as it reflects beliefs about the possibility of transforming broader social conditions and structural constraints through collective effort. Psychological hope, by contrast, is primarily concerned with individual, goal-directed thinking and personal pathways to desired outcomes.

### Intersectionality

For the purposes of this investigation, we adopt an intersectional approach to CAR. Our intersectional lens attends to the gendered harms WOC face, including gendered racism. Defined by critical race scholar Philomena Essed (11), gendered racism speaks to the interaction of both ethno-racism and sexism that impact WOC. This intersectional approach to understanding oppression emphasizes the importance of exploring how power differences occur simultaneously within both domains of gender and race. Importantly, gendered and racial oppression extends beyond binary categories. As such, nonbinary participants were included in the study to better capture the experiences of all individuals impacted by gendered-racialized oppression. However, given the limited number of non-binary participants in the present study, the language of “Women of Color” (WOC) is used to reflect the primary composition of the sample and the subsequent analyses presented. WOC, holding minoritized identities in both gender and racial categories, are thus subject to the unique harms of gendered racism that are not experienced by Men of Color or White women.

Restrictions to a person’s personal autonomy have been shown to be associated with negative mental health outcomes (12). However, marginalized communities, such as WOC, often face multiple and intersecting group-level restrictions to autonomy such as restrictions in healthcare decisions, political action, and cultural practice. For example, across the history of the United States, Black, Latina, and Indigenous women were disproportionately targeted for forced sterilization in ways that White women were not (3). Beyond reproductive autonomy, Black women have been specifically harmed by natural hair bans in the workplace, a form of gendered racism. Greene (13) noted that Black women have consistently fought against workplace policies that restricted the wearing of culturally significant hairstyles in ways that distinctly targeted Black women, over other women or People of Color. While these restrictions to autonomy impact individual WOC, they also occur at the group level, impacting WOC collectively. Although the impact of restrictions to personal autonomy has been extensively studied in psychology, little is known about restrictions to collective autonomy. The purpose of the present study was thus to explore the influence of the awareness of systemic restrictions on WOC.

#### Defining collective autonomy restriction

At its core, collective autonomy refers to a person’s sense that the social group(s) they belong to has the freedom to define and practice their own culture/identity independently of the larger society (14). CAR thus reflects policies and practices that limit a group’s human rights and freedoms, such as laws governing who is allowed to marry or rules limiting the pronouns people use. CAR against women is a form of gendered harm because it limits women’s autonomy to make decisions in their own individual and collective interests and reinforces discriminatory practices which impact marginalized women the most. For example, France’s controversial hijab ban represents the gendered-anti-Muslim restriction of women’s autonomy (15).

CAR is a relatively new construct in the field, but emerging research indicates that it is related to a range of psychological and behavioral processes among marginalized people cross-culturally. For example, findings have indicated that increased sense of CAR was associated with lower levels of psychological well-being among cultural groups in the United States and India, and LGBTQ+ individuals (14, 16). Directly related to this study, Kachanoff and colleagues (17) recently investigated the link between the awareness of CAR and support of collective action and lower endorsement of system justification among Black Americans. CAR was significantly associated with wanting and pursuing greater social power through collective social action and with greater opposition to system-justifying myths (i.e. Protestant Work Ethic). While some of these studies have explored the impact of the awareness of CAR on people who are multiply marginalized, we were unable to locate a study that explicitly adopted an intersectional approach that considered collective restrictions grounded in gendered racism.

#### Connecting CAR to critical consciousness

Throughout history, WOC have recognized that their own rights and freedoms are deeply tied to those of others; with this understanding, they have consistently participated in broader struggles for justice. Their actions have often been fueled by a belief that change is possible (what we call radical or collective hope), an awareness of the oppressive conditions shaping their lives, and a commitment to working alongside others to improve their circumstances. In this study, we set out to explore these core conditions—not only to understand or explain CAR among WOC, but also to ask whether recognizing these restrictions is associated with a motivation and willingness to take action (critical consciousness), and how hope may shape this process.

Critical consciousness (CC) describes the development of critical knowledge and analysis of one’s own social conditions and the ways one can make change (18, 19). Conceptualized by Brazilian philosopher and educator Paulo Freire (19), CC involves an ongoing process of learning and developing awareness of oppressive social, political, and economic structures. By addressing the root causes of oppression, CC is considered by many critical scholars as a necessary condition for healing or liberation from oppression. Freire emphasized that through CC, individuals actively participate in their own liberation by engaging with their communities rather than remaining passive subjects of imposed systems. Through the process of CC raising, marginalized communities increase their awareness and knowledge of oppression while also positioning themselves within these systems and working to identify modes of action.

Scholarship on CC is growing. Building on Freire’s (19) conceptualization, Watts and colleagues (20) proposed a three-dimensional approach to CC, incorporating critical reflection, political efficacy or critical agency for change, and critical social action. Critical reflection involves a critical analysis of oppressive systems. Initially described as political efficacy, and sometimes described as critical agency (18, 21), critical motivation is the perceived capacity to effect sociopolitical change. Critical action involves participation in social action. It is through critical reflection that one can then consider how they may be a part of changing these systems and ultimately engage in those actions. Importantly, CC is not a linear journey towards a final stage of consciousness, rather it is a constant and iterative process. CC development can be facilitated throughout the lifetime by varying methods, including learning one’s cultural history, exploring methods of resistance, and community building (18, 21, 22). Literature regarding the potential effects of CC on psychological well-being is growing. A review of CC scholarship on mental health noted that while CC is associated with more positive mental health outcomes with youth, its effects among adults are mixed (23). Among sexual minorities, for example, research has suggested that CC, specifically critical reflection, predicts lower internalized oppression and increased community connectedness while being positively associated with emotional distress (24).

In the present study, CAR is conceptualized as a specific form of critical reflection—one that centers awareness of collective restriction within broader structural contexts; this is consistent with prior CC research suggesting that critical reflection is what inspires a stronger sense of critical motivation and a greater willingness to engage in collective action (25). For example, Kachanoff and colleagues (17) found that among Black Americans, the perception of increased CAR was associated with efforts to challenge social hierarchy through collective action. Further, research has connected experiencing discrimination, a similar construct to CAR, with critical motivation (26) and critical action (21). Additionally. Hope and colleagues (27) found that critical reflection was linked to both critical motivation and action among a sample of Black adolescents with greater experiences of racism-related stress. Therefore, conceptual reasoning suggests that CAR is an indicator of critical reflection and, as such, is likely to be associated with the other two CC dimensions (motivation and action).

#### Complicating the CAR and CC association: do psychological and radical hope matter?

For the present study, we (a) explored the associations between CAR and critical motivation and critical action and (b) examined the role of two types of hope as potential mediators that may account for indirect effects on these pathways. The psychological concept of hope as both a cognitive tool and an emotional state or trait is well-studied (28, 29). Differentiated from simple wishful thinking or optimism, psychological hope entails not only the cognitive belief that one has the tools, ability, and sense of agency to achieve goals in the future (28). This construct has been associated with varying measures of positive mental health and development among WOC (30–32).

While agency is a core component of hope and is similar to our established understanding of collective autonomy, they are different concepts. For our purpose, it is essential to make this distinction clear. Collective autonomy refers to the ability of a group to engage with their culture; agency refers to the power an individual or a group has to take action (33, 34). Specifically, agency reflects the belief that one is able to enact change--hence its relevance and use in varying theories of hope (7, 28).

Emerging research documents an association between hope and critical motivation and critical action more specifically (35, 36). For example, in a longitudinal study of Youth of Color, Suzuki et al. (37) found that greater levels of psychological hope was connected to increased trajectories of both critical motivation and action. Thus, youth who were more likely to believe good things will happen for themselves in the future, and they were also more likely to show increases in a critical sense that they can take action that will make a difference to create change (motivation) and actually engage in such action over time.

Similar to traditional imaginings of autonomy, psychological hope is often conceptualized as tied to an individual’s sense of their own agentic abilities. For Communities of Color, collectivism rather than individualism is a foundational cultural value. During times of systemic and seemingly insurmountable atrocities like mass deportations, attacks on queer, immigrant, and trans communities, bans on abortion rights, and rises in overt White supremacist actions, psychological hope alone may not address the impact of hope with and for one’s community. Given the important role of the collective in marginalized communities and well-being, a construct of hope that honors the collective is paramount.

A number of scholars have speculated about the association between collective or radical hope and taking action to improve the lives of marginalized groups (7, 38). Radical hope entails envisioning a collective more just future despite seemingly insurmountable odds. During the uprisings of June 2020, the U.S. saw radical hope across the Black community and allies as they fought against seemingly unending racism and police brutality. The pain continued but hope for change is what motivated people to protest. Rooted in intersectionality, liberation, and Black feminist thought, radical hope has two major components: *Collective Orientation* and *Faith and Agency*, and five pathways: *Embracing Racial Pride*, *Meaning Making and Purpose, Resisting Racism*, *Envisioning Possibilities*, and *Valuing Self* (7, 39). The pathways describe ways POC may find to foster radical hope, while the core components speak to the constructs that make up radical hope. Collective orientation requires remembering those who came before us and the lessons they taught us while holding ourselves responsible for continuing that legacy. Faith and agency involve the belief that a future is possible and prioritize the engagement with action to make them. The empirical research on radical hope is in its infancy, but emerging qualitative studies provide support for the psychology of radical hope framework (39) and indicate that radical hope and CC are central to capacity building among formerly incarcerated Black and Latino men (40).

### Rationale and purpose

The erosion of women’s rights and the increasing restrictions on reproductive freedom and weakened legal protections against gender-based violence, which disproportionately impact WOC, are contemporary examples of CAR. Being aware of these restrictions (critical reflection), believing in one’s capacity to enact change (critical motivation), and taking action to address inequalities are essential in improving long-term mental health and sustaining social movements to bring about change in society (critical action). There is conceptual and emerging research suggesting that those with greater awareness of CAR are likely to endorse a sense of efficacy or motivation that action can make a difference and getting involved in individual and collective action to create change. There is little research specifically on WOC experiences with CAR and the remaining two dimensions of CC.

In the present study, we were particularly interested in examining the role of hope in understanding the association between CAR (critical reflection) and critical motivation and action. Hope is both a goal-directed psychological orientation and a collective attitude that can counter feelings of despair and demobilization. Research indicates both psychological, collective, and a combination of the two types of hope predicts action. Wlodarczyk et al.’s (41) and Phoenix’s (42) findings, for example, connected collective hope to action. We build on the work of Wlodarczyk et al. who found that a sense of group unity was associated with critical action through the sequential processes of increased critical efficacy (motivation) and collective hope among adults in Spain. Conceptually applied to the present study, hope could feasibly function as a motivational force that transforms awareness of collective restriction into agentic participation. Based on the available literature, we hypothesized that the association between CAR and critical action would be explained through serial pathways in which higher motivation predicted greater hope, which in turn predicted critical action.

## Materials and Methods

### Participants

Data were collected as part of a larger study on radical hope. The final sample consisted of 408 participants, 405 of whom provided demographic information. Participants ranged in age from 18 to 82 years (*M* = 33.9, *SD* = 11.87). 47.4% (*n* = 192) were African American or Black, 22.2% (*n* = 90) Asian American or of Asian descent, 20.0% (*n* = 81) Latinx of any racial group, 7.7% (*n* = 31) bi-ethnic or multiracial, 2.5% (*n* = 10) American Indian or Alaskan Native, 0.2% (*n* = 1) Middle Eastern/North African (MENA). A majority of participants self-identified as women (92.6%; *n* = 376) and significantly fewer identified as non-binary, agender, or Two-Spirit (7.2%; *n* = 29). Information regarding participant education level, immigration, and generational status was also collected. See **Table 1** for a full report of participant demographic information.

**Table 1:** Demographic data.

Characteristic	N = 408
REC identity	(N = 405)
African American or Sub-Saharan African	192 (47.4%)
Asian or Asian American	90 (22.2%)
Hispanic or Latino/Latina/Latinx	81 (20.0%)
Native American/American Indian	10 (2.5%)
Middle Eastern, Southwest Asian or North African	1 (0.2%)
Bi-ethnic or Multiethnic	31 (7.7%)
Gender	(N = 406)
Women	376 (92.6%)
Nonbinary/Gender non-conforming/Other gender identity	29 (7.2%)
Prefer not to say	1 (0.2%)
Sexual orientation	(N = 406)
Heterosexual	276 (68.0%)
Lesbian	18 (4.4%)
Bisexual	83 (20.5%)
Other	24 (5.9%)
Prefer not to say	5 (1.2%)
Religion	(N = 335)
Agnostic	58 (17.3%)
Atheist	39 (11.6%)
Buddhist	13 (3.9%)
Christian	201 (60%)
Hindu	2 (0.6%)
Muslim	14 (4.2%)
Wiccan/Pagan/NeoPagan	2 (0.6%)
Other	6 (1.8%)
Generational status	(N = 404)
First generation	68 (16.8%)
Second generation	146 (36.1%)
Third+ generation	178 (44.1%)
Not Listed	12 (3.0%)
Education	(N = 406)
Some high school	5 (1.2%)
High school graduate	60 (14.8%)
Some college	80 (19.7%)
Associate’s degree	38 (9.4%)
Bachelor’s degree	142 (35.0%)
Some postgraduate	10 (2.5%)
Master’s degree	63 (15.5%)
Ph.D., law, or medical degree	7 (1.7%)
Other advanced degree beyond master’s	1 (0.2%)
Geographic region	(N = 405)
Northeast	57 (14.1%)
South	176 (43.4%)
Midwest	49 (12.1%)
West	123 (30.4%)

### Measures

To facilitate interpretation and analyses across scales with differing items, each measure, outside of demographics, was scored using mean scores rather than sum scores.

Demographic. A 13-item demographic questionnaire was constructed for the study to obtain information about participants’ race, ethnicity, gender, age, political affiliation, immigration status, generational status, education level, religious identities, and sexuality.

Collective Autonomy Restriction (an indicator of critical reflection). The 8-item Collective Autonomy Restriction scale (CAR; 14) was used to explore group members’ perception that other groups have tried to restrict and control the identity and expression of their ingroup. In this study, perceived restrictions in collective autonomy were assessed with respect to the racial and/or ethnic and gender identities that participants self-generated and considered as their “core” group. The following prompt was provided to participants: “For the following questions, please consider your specific ethno racial gender identity. That is, the group you refer to naturally when people ask you what your ethno-racial and gender identity is, and you reply ‘I am x. In the text box below, please describe your ethno racial and gender identity. (For example, I am a Black Woman, a Non-binary Asian person, a Mexican Trans Latina).” Participants provided responses on a 7-point Likert-type response scale ranging from 1 (*strongly disagree)* to 7 (s*trongly agree*) for 8 items (e.g. “Other groups have tried to control us” and “Other groups impose aspects of their culture onto our culture”). Higher sum CAR scores indicate greater agreement of restriction of ingroup autonomy or perceived limits of one’s social group self-determination. Confirmatory factor analyses by Kachanoff and colleagues (14) found that CAR items loaded onto a single construct, distinct from personal autonomy. Moreover, findings have highlighted its negative association with personal autonomy (14, 16) and system justification among Black Americans (17). Internal consistency estimates from existing literature were acceptable ranging from 0.92 (14) to 0.94 (17); The coefficient alpha estimate in the current study was also excellent (*α* = 0.96).

Psychological Hope. The widely used 12-item Adult Hope Scale (AHS; 43) was administered to assess personal or psychological hope. The AHS is comprised of two subscales: agency (e.g., “I energetically pursue my goals”) and pathways of thinking (e.g., “I can think of ways to get out of a jam”) as well as 4 are distractor items (e.g. “I feel tired most of the time”). Participants respond to the items using an 8-point Likert-type scale ranging from 1 (*definitely false*) to 8 (*definitely true*), with higher sum scores indicating greater endorsement of hope traits. AHS has been validated across diverse ethnoracial and cultural groups (Cronbach alphas of 0.81 to 0.92 across several studies; 44–46) and genders (47). Confirmatory factor analysis has supported this two factor model of hope (48). Research has also demonstrated measurement invariance of AHS across ethnoracially and gender diverse populations (49). Internal consistency in the present study was 0.90.

Radical Hope. The 21-item Radical Hope Scale (RHS; 50) was used to assess collective hope or the belief that greater racial justice in the future is possible. The items are rated on a 6-point Likert-type scale, ranging from 1 (*strongly disagree*) to 6 (*strongly agree*) and includes assessment of collective radical hope (*“*People in my community can create a more racially just world”) and personal radical hope (“Knowing how my community has resisted racial oppression motivates me to fight for racial justice”). A total radical hope score is a summed average score. Findings from exploratory and confirmatory factor analyses among POC adults indicated that a three factor model was a good fit to the data and that the RHS total was associated with greater levels of meaning in life, life satisfaction, psychological hope, and joy (50). Internal consistency for the present sample was 0.93.

Critical Consciousness. Critical Motivation and Critical Action were measured by the Short Critical Consciousness Scales (ShoCCS; (51). The ShoCCs is a 13-item scale that assesses three dimensions of CC: Critical Reflection (e.g., “Certain racial or ethnic groups have fewer chances to get good jobs”), Critical Motivation (which is comparable to the description of agency in the introduction) (e.g., “It is important for young people to know what is going on in the world”), and Critical Action (e.g., “Participated in a civil rights group or organization”). For the purposes of the present study, CAR was administered as an indicator of CC reflection and thus the ShoCCS reflection subscale was not used in the current investigation. For the Critical Motivation subscale, participants respond to the items using a 6-point Likert-type scale ranging from 1 (*strongly disagree*) to 6 (*strongly agree*). The Critical Action subscale items ask respondents to report how often they were involved with an activity over the last year from 1 (*never did this*) to 5 (*at least once a week*). ShoCCs scoring typically indicates that higher sum scores on each subscale indicated higher levels of the given scale. For the present study, given that the Critical Action subscale utilized different response scales and item numbers than the Critical Motivation subscale, average composite scores were utilized to facilitate more intuitive comparison across subscales. Importantly, this transformation does not affect the pattern of results. The ShoCCS has been demonstrated to have strong psychometric properties based on findings from a confirmatory factor analysis and strong correlations with the long-form critical consciousness (51). Internal consistency for the scale in previous studies was strong at 0.88 (Critical Motivation), and.85 (Critical Action). Coefficients alpha across the two subscales for the present sample was excellent: Critical Motivation (α= .73), and Critical Action (α= .92). Importantly, Diemer and colleagues (51) clarified that the scale was developed for use with both youth and adult populations. Research has continued to explore its utility with emerging adult populations (23).

### Procedure

Prior to data collection, approval from the Institutional Review Board (IRB) for human subjects was obtained. Participants were recruited via Prolific, which allowed researchers to obtain a diverse sample of participants who respond to surveys consistently and provide quality responses (52). Participants were screened for their ethno-racial and gender identity. Three items throughout the full survey asked about gender; participants who consistently identified as women or non-binary across the three questions were included in the study. Respondents were paid $6.00 for their participation. After signing the consent form and completing the screener items (ethno racial identity and age), participants were presented with the measures (i.e.,. RHS, AHS, CARS, ShoCCS) in random order, followed by the demographic questions. Data were collected between December 2024 and February 2025.

The initial sample included 435 self-identified women or non-binary people. Duplicate cases, cases with over 80% missing data, participants who did not consistently identify as women or non-binary, and participants who did not pass attention checks were removed during data cleaning. Twenty-seven cases were deleted, and the remaining 408 participants were utilized for the study. Participants identifying as women or non-binary on Prolific were prompted to complete the CAR scale.

### Data analytic strategy

Data analyses were conducted using SPSS version 31.0 (24) and Mplus 8.8 (1998-2017). All variables were screened for missing data and outliers. Our final sample consisted of 408 participants. Across the relevant study variables, there was minimal missingness (0.5% - 4.7%). Findings from Little’s Missing Completely at Random (MCAR) test suggest data were missing completely at random (*χ*² = 26.40, *df* = 26, *p = .*44). The assumptions of normality were also met as indicated by skewness and kurtosis values between ± 2 (53).

#### Stage 1: descriptive statistics and bivariate analyses

Descriptive statistics, including means and standard deviations were computed using SPSS (54) for all study variables. Pearson correlation coefficients were assessed to explore associations between study variables. Effect sizes were interpreted using Cohen (55) guidelines (small: *r* = 0.10, medium: *r* = 0.30, large: *r* = 0.50).

#### Stage 2: serial mediation analysis

Serial mediation using structural equation modeling (SEM) was tested using Mplus 8.8 (1998-2017). Serial mediation analysis enabled us to account for the complicated processes of developing critical consciousness by including multiple non-consecutive mediating variables (Critical Reflection → Critical Motivation → Hope → Critical Action). Statistical significance was evaluated using 95% confidence intervals from the delta method. Most variables in the structural equation model were specified as observed variables, with the exception of Critical Action, which was modeled as a latent construct. Because Critical Action consisted of ordinal items we used Weighted Least Squares Mean and Variance (WLSMV) estimation as it is often the default and best practice for use with ordinal variables (56).

Serial mediation is considered supported when the sequential indirect effect is statistically significant. In the present model, serial mediation is supported when CAR influences Critical Action through its effects on Critical Motivation and then hope (RHS and AHS). The present model estimated the total effect of CAR on Critical Action (path c), the total effect controlling for both hope mediators (RHS and AHS) (path c’) and three indirect pathways. All pathways first examined the impact of collective autonomy restriction (CAR) on Critical Motivation (path a_1_). The first pathway examined the subsequent impact of Critical Motivation on Critical Action (path b_1_). The second pathway examined the effect of Critical Motivation on Radical Hope (path d_21_) and then on Critical Action (path b_2_). The final pathway examined the effect of Critical Motivation on Psychological Hope (path d_31_) and then on Critical Action (path b_3_). This serial mediation model allowed us to capture the process of experiencing awareness of collective restriction on one’s motivation for change, which then influences a person’s sense of hope and finally their engagement in social action. Full mediation is indicated by significant indirect effects and non-significant direct effects. Partial medication is indicated by significant direct and indirect effects. Several standard fit indices were used to evaluate model fit, including chi square, the Comparative Fit Index (CFI), the Tucker Lewis Index (TLI), and the Root Mean Square Error of Approximation (RMSEA).

## Results

### Descriptive statistics

Descriptive statistics and Pearson’s correlations are available in **Table 2**. There were small to large positive correlations between the CAR and study variables, ranging from (.24) to (.57). Notably, RHS had a large positive correlation with Critical Motivation (*r* = .57) and moderate correlation with CAR (*r = .*37). Means for the present sample suggest high levels of Collective Autonomy Restriction (*M* = 5.48, *SD* = 1.24) and Critical Motivation (*M* = 5.13, *SD* = 0.74). Mean scores for Critical Action were low (*M* = 1.77, *SD* = 0.94). Mean levels of both measures of hope were also moderately high (AHS: *M* = 5.84. *SD* = 1.25; RHS: *M* = 4.64, *SD* = 0.68). Ranges for each variable measure are presented in **Table 2**.

**Table 2:** Descriptive statistics with person correlations and cronbach’s alphas for variables of interest.

Variable		*N*	1	2	3	4	5	*M*	*SD*
1	CAR	404	--					5.48	1.24
2	RHS	389	0.37**	--				4.64	0.68
3	AHS	406	0.04	0.28**	--			5.84	1.25
4	Critical Motivation	406	0.34**	0.57**	0.19*	--		5.13	0.74
5	Critical Action	405	0.08	0.30**	0.29**	0.24**	--	1.77	0.94

*p < 0.05, **p <.001; CAR, Collective Autonomy Restriction (CAR possible range is 0 to 7); RHS, Radical Hope Scale (RHS possible range is 0 to 6); AHS, Adult [Psychological] Hope Scale (AHS range is 0 to 8]; Critical Motivation range is 0 to 6; Critical Action range is 0 to 5.

### Serial mediation analysis

**Figure 1:**
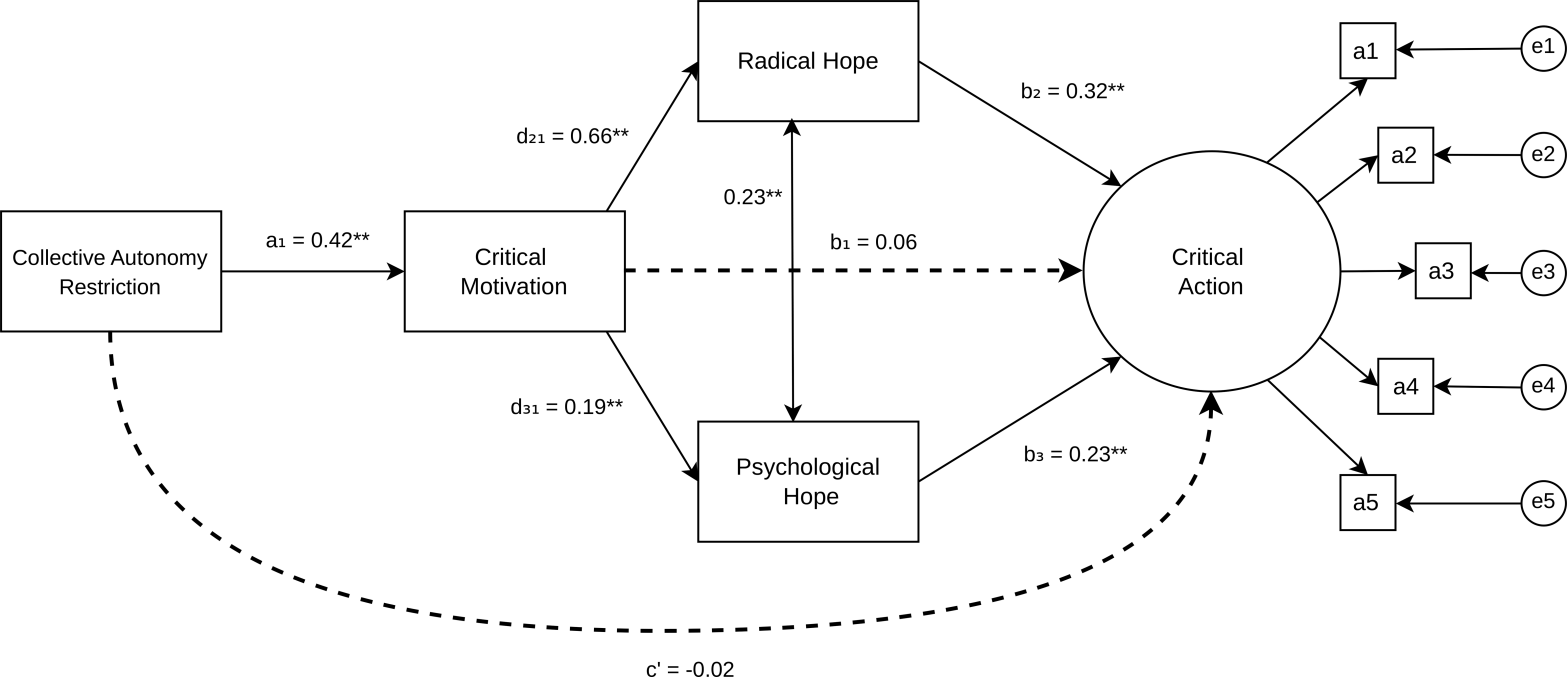
Serial mediation model of CAR on critical action through critical motivation and hope with standardized path coefficients. Dashed lines represent non-significant direct effects. The double-sided solid arrow represents the covariance between radical hope and psychological hope mediators. *p < .001.

To explore the associations between CAR, Critical Motivation, hope and Critical Action, we conducted a serial multiple mediation model. **Figure 1** presents the proposed model's standardized path coefficients. See **Table 3** for the detailed path findings. Our model was well fit to the data. CFI and TLI were .99 and SRMR was .03, suggesting good model-data fit (57). The RMSEA of .08 suggests a marginally appropriate fit (58). Factor loadings on latent variables were all highly loaded (> 0.80). The chi-square test was significant (χ² = 81.82, df = 23, p <.001) though this is expected given our large sample size. Overall, fit indices suggest appropriate model fit.

**Table 3:** Direct, indirect, and total effects of collective autonomy restriction on action through motivation and hope.

					95% CI		
Direct effects	B	β	S.E.	*p*	Lower bound	Upper bound	*R²*
CAR → Motivation (a₁)	0.24	0.42	0.04	<.001	0.34	0.50	0.17
Motivation → Radical Hope (d₂₁)	0.60	0.66	0.03	<.001	0.60	0.72	0.43
Motivation → Psychological Hope (d₃₁)	0.33	0.19	0.05	<.001	0.10	0.28	0.04
Motivation → Action (b₁)	0.26	0.07	0.09	0.45	−0.11	0.24	--
Radical Hope → Action (b₂)	1.40	0.32	0.09	<.001	0.15	0.50	--
Psychological Hope → Action (b_3_)	0.53	0.23	0.05	<.001	0.13	0.33	--
Radical Hope ↔ Adult Hope	0.14	0.23	0.04	<.001	0.15	0.32	--
CAR → Action (c’)	−0.05	−0.02	0.06	0.70	−0.14	0.09	0.23
Indirect effects	B	β	S.E.	*p*	95% CI		*R²*
					Lower bound	Upper bound	
Total Indirect	0.31	0.13	0.03	<.001	0.08	0.19	--
CAR → Motivation → Action (a₁b₁)	0.06	0.03	0.04	0.45	−0.04	0.10	--
CAR → Motivation → Radical Hope → Action (a₁d₂₁b₂)	0.20	0.09	0.03	0.001	0.04	0.14	--
CAR → Motivation → Psychological Hope → Action (a₁d₃₁b₃)	0.04	0.02	0.01	0.004	0.01	0.03	--
Total effect	B	β	S.E.	*p*	95% CI		*R²*
					Lower bound	Upper bound	
CAR → Action	0.26	0.11	0.06	0.05	0.00	0.22	--

B, unstandardized coefficient; β, standardized coefficient; SE, standard error; CI, confidence interval; Radical Hope ↔ Psychological Hope reflects the covariance.

#### Direct effects

The direct effect of CAR on Critical Motivation (path a_1_) was significant, suggesting that increased CAR was associated with increased Critical Motivation (β = .42, *SE* = 0.04, *p* <.001, 95% CI [0.34, 0.50]). Critical Motivation appeared to have a significant effect on both RHS (path d_21_; β = .66, SE = .03, *p* <.001, 95% CI [0.60, 0.72]) and AHS (path d_31;_ β = .19, SE = .05, *p* <.001, 95% CI [0.10, 0.28]). Covariances between RHS and AHS were significant (β = .23, SE = .04, *p* <.001, 95% CI [0.15, 0.32]). The direct effects of both mediators on Critical Action were significant such that increased RHS (path b_2;_ β = .32, SE = .09, *p* <.001, 95% CI [0.15, 0.50]) and increased AHS (path b_3_; β = .23, SE = .05, *p* <.001, 95% CI [0.13, 0.33]) were associated with greater Critical Action. Notably, the direct effect of CAR to Critical Action (path c’) was not significant (β= -.02, SE = 0.06, *p* = 0.70, 95% CI [-0.14, 0.09]). Moreover, CAR was non-significantly associated with Critical Action (path c) (β= .11, SE = 0.06, *p* = 0.05, 95% CI [0.00, 0.22]). Together, the statistically non-significant direct and marginally non-significant total effects suggest full mediation, operating through mediation pathways.

#### Indirect effects

Consistent with our hypothesis, the total indirect effect of CAR on Critical Action was significant (β = .13, SE = .03, *p* <.001, 95% CI [0.08, 0.19]), through the hypothesized serial mediation. Three proposed pathways were explored. The first proposed pathway from CAR to Critical Action through Critical Motivation alone was not significant (β = .03, SE = 0.04, *p* = 0.45, 95% CI [−0.04, 0.10]) suggesting that Critical Motivation alone does not mediate the association between CAR and Critical Action. Rather, our second and third pathways suggest our hypothesized serial mediation effects of both AHS and RHS on Critical Action were each significant.

In the second pathway, increased CAR was associated with increased Critical Motivation which was associated with increased RHS (d_21_; β = .66, p <.001, 95% CI [0.60, 0.71]) and subsequently associated with increased Critical Action (b_2_; β = .32, p <.001, 95% CI [0.15, 0.50]). The overall second indirect pathway was significant (β = .09, *SE* = .03, *p* <.001, 95% CI [.04,.14]). In our third pathway, increased CAR was associated with increased Critical Motivation which was associated with increased AHS (d_31_; β = .19, *SE* = .05, *p* <.001, 95% CI [.10,.28]), and subsequently associated with increased Critical Action (b_3_: β = .23, *SE* = .05, *p* <.001, 95% CI [.13,.33]). The overall third indirect pathway was significant (β = .02, SE = .006, *p* = .004, 95% CI [.01,.03]). However, the sequential mediation pathway through RHS predicting Critical Action was stronger than the pathway through AHS.

## Discussion

The purpose of this study was to explore the connection between awareness of restrictions to collective autonomy among WOC and critical motivation and action, and to analyze the potential role of hope as an influencing factor. We were particularly interested in two dimensions of hope: psychological and radical hope. Findings add to the growing body of research on the complex relations among the three components of critical consciousness, particularly the idea that critical consciousness may develop sequentially rather than occurring simultaneously (59), and on the role of hope as a mechanism linking critical reflection and critical motivation to critical action (41). Specifically, we found support for our hypothesis that the association between CAR and critical action operates through serial pathways involving critical motivation and hope, with the strongest pathway occurring through radical hope. Existing literature has noted the relevance of experiencing oppression in the development of agency or commitment to resistance (26, 60). In the present study, CAR was found to have a strong positive association with critical motivation, such that a person who reported awareness of increased group-level restriction was more likely to feel motivated and committed to enacting change. Still, while awareness of collective autonomy restriction was associated with higher levels of critical motivation, neither of these associations directly translated into actions. Research describing the barriers to engagement in activism describe possible reasons for the low critical action among participants, including the belief their actions may not make a difference, lack of education regarding how to get involved, and fear for their safety (61, 62). Ultimately, the association between CAR and critical action and the relation between critical motivation and critical action was significant but complicated in that it was explained through hope. Essentially, hope played a significant role in linking the three components of critical consciousness.

In support of our hypothesis, CAR was associated with greater critical motivation to take action, which in turn was associated with increased radical hope and, ultimately, greater reports of critical action. Radical hope thus served as an explanatory variable. Our findings suggest that WOC who had greater levels of belief that they should and can make a change in society expressed greater hope for justice for their community, despite seemingly insurmountable odds, were also more likely to show higher levels of belief they can make a change. It was this sense of hope that was associated with participants reporting engagement in actions to address injustices in society. These findings align with the conceptual frameworks of both radical hope (39) and critical consciousness (63), as well as research regarding hope among Youth of Color (37). Notably, our findings extend the work of Suzuki and colleagues (2023) by highlighting that radical hope uniquely mediated the association in all three dimensions of critical consciousness. CAR, radical hope and critical motivation and action are each intrinsically tied to an awareness and understanding of oppression. However, awareness of group restriction alone nor motivation to take action is not enough to enact change. Rather, radical hope appears to be a key factor to take action to help create social change and to take action to address injustices in society.

Consistent with extant literature, psychological hope was positively associated with action. However, the magnitude of the role psychological hope played in understanding the association between CAR and CC was less than that of radical hope. In this study, radical hope played a distinct and comparatively greater role than psychological hope. Constructs associated with critical social analysis (i.e., CAR) did not appear to align as clearly with psychological hope. One possible explanation for this finding is that psychological hope centers individual agency and motivation but does not account for broader systemic or community-level factors. Ultimately, psychological hope does not incorporate the critical social analysis needed to understand injustice and/or anticipate the development of critical consciousness. Meanwhile, radical hope, like CAR and critical motivation and critical action, is a construct rooted in understanding social groups and oppression rather than individual cognition. As such, radical hope may be uniquely important to understanding the role of restrictions to collective autonomy in shaping critical consciousness and long-term wellbeing for WOC.

### Limitations

Although the findings extend the emerging CAR and CC research, there are several noteworthy limitations to consider. These exploratory findings offer some insight into the potential role of radical hope in explaining the association between CAR (critical reflection) and critical motivation and action. Given that the data are cross-sectional, the findings are correlational and claims cannot be made about directionality. In the present study we chose to examine radical hope as a mediator based on previous conceptual and empirical research. However, a case could also be made for critical motivation and action serving as mediators between the association between CAR and radical hope. Future research should collect longitudinal data and investigate reciprocal effects and competing models among CAR, hope, and critical motivation and action. Second, in order to explore CAR, participants were asked to first consider their ethno racial and gender identity (e.g., “I am a Native American woman”). While this information provided some context to their responses, further information, such as asking them to recall a specific instance of restriction, may have been helpful in ensuring their understanding of the question. By asking for ethno-racial-gender identity and allowing participants to respond to items without prompting about a specific event of restriction, it may have allowed participants to consider their lifetime experience of restriction rather than connecting their awareness of restriction to a particular policy or event.

A third potential limitation is related to the recruitment of participants. While Prolific allowed us to recruit a group of ethno-racially diverse participants, our study reflected some bias as more than half of the participants obtained at least a bachelor’s degree, an 18% increase compared to the national average for WOC. As some research has indicated a positive association between level of educational attainment and critical consciousness (64), the high level of education in our sample may have also skewed critical consciousness scores. Finally, while the present study consisted of an ethno racially diverse group of participants, the sample was not large enough to detect ethno racial group-level patterns. By obtaining a larger sample size, further analysis of these differences may have been possible and provided a more robust analysis of the impact of oppressive policies on varying ethno-racial groups.

## Future research directions

This study expands existing research regarding autonomy restriction, critical consciousness, and hope. Further research may seek to clarify the scope of collective autonomy restriction as an indicator of critical reflection. As attacks on the autonomy of marginalized groups continue to rise in the U.S., additional research on CAR may highlight the impacts of specific attacks on autonomy. This research may provide fertile ground for updated definitions of autonomy that address the personal and collective nature of bodily autonomy. For example, given the increased burden of anti-abortion policies on Black women in particular, future research may explore possible ethno racial group differences in the association of collective autonomy restriction on psychosocial outcomes.

The field of psychology would benefit from research exploring the use of the varying dimensions of critical consciousness (including awareness of CAR as an indicator of critical reflection) on psychological healing. While research regarding the impact of CC on psychological well-being is limited, some findings have indicated that CC acts as a buffer against psychological distress for WOC (65). Given the continued collective stressors marginalized communities face, from threats of mass deportation to gender-affirming care bans and an increase in hate crimes, understanding how they may buffer these harms and provide protective support is paramount. In this study, only CAR was specific to WOC and reproductive justice. Future research should extend the current study by using reproductive justice indicators of critical motivation, radical hope, and critical action. Additionally studies are needed to explore the role of CC around reproductive justice on psychosocial well-being. Finally, the current findings also elucidated the unique role radical hope plays in the critical consciousness process. Marginalized people in the U.S. are experiencing historic levels of harm and vitriol, and exploration of psychological tools to buffer these harms is critical. Given the need for change and resistance and the power of agency in engaging in social change, future research should investigate radical hope as a therapeutic consideration. Longitudinal designs are essential in this line of research to test the causal relationships and developmental processes underlying critical consciousness, as well as the role of radical hope in this process.

### Implications for practice

The results of this study have significant implications for clinical practice and intervention. Given that CAR was positively associated with critical motivation and radical hope, which in turn was related to taking critical action to address injustices in society, addressing these restrictions in the therapeutic space is warranted. These findings align with research that emphasizes the importance of addressing societal factors with our clients (66–68). As restrictive and harmful anti-abortion, anti-trans and anti-immigrant policies proliferate, it is an ethical imperative that clinicians maintain their knowledge and education regarding political and cultural shifts that may impact their clients. Working to externalize the harms of an oppressive cultural milieu, helping clients explore the power they have to impact change, and fostering a sense of hope that change is possible may serve to buffer its harms (67, 69). In light of the present study, therapists may invite clients to describe how these socio-political policies and cultural shifts have impacted them.

Some existing research has found that critical consciousness, via critical action, was associated with greater life satisfaction (70). The findings of the present study suggest that radical hope was a predictor of critical consciousness across all of its dimensions, including critical action. In light of our findings, clinicians should explore ways to foster collective action through the development of radical hope. Brainstorming with clients what hope means to them, how they can practice radical hope in their daily lives, and helping them to connect hope into a better tomorrow to direct action. 

## Data Availability

The raw data supporting the conclusions of this article will be made available by the authors, without undue reservation.
